# Learning Needs of Pharmacists for an Evolving Scope of Practice

**DOI:** 10.3390/pharmacy7040140

**Published:** 2019-09-25

**Authors:** Zubin Austin, Paul Gregory

**Affiliations:** 1 Leslie Dan Faculty of Pharmacy and the Institute for Health Policy, Management and Evaluation—Faculty of Medicine, University of Toronto, Toronto, ON M5S 3M2, Canada; 2 Leslie Dan Faculty of Phamacy, University of Toronto, Toronto, ON M5S 3M2, Canada; streetsoccercanada@mac.com

**Keywords:** pharmacy practice, continuing professional development, scope of practice, quality assurance, maintenance of competency

## Abstract

Around the world, changes in scope of practice regulations for pharmacists have been used as a tool to advance practice and promote change. Regulatory change does not automatically trigger practice change; the extent and speed of uptake of new roles and responsibilities has been slower than anticipated. A recent study identified 9 pre-requisites to practice change (the 9Ps of Practice Change). The objective of this study was to describe how educationalists could best apply these 9Ps to the design and delivery of continuing professional development for pharmacists. Twenty community pharmacists participated in semi-structured interviews designed to elicit their learning needs for scope of practice change. Seven supportive educational techniques were identified as being most helpful to promote practice change: (i) a coaching/mentoring approach; (ii) practice-based experiential learning; (iii) a longitudinal approach to instructional design; (iv) active demonstration of how to implement practice change; v) increased focus on soft-skills development; (vi) opportunities for practice/rehearsal of new skills; and (vii) use of a 360-degree feedback model. Further work is required to determine how these techniques can be best applied and implemented to support practice change in pharmacy.

## 1. Introduction

The profession of pharmacy continues to evolve in order to meet the changing needs of patients [1]. Internationally, scope of practice change has been an important and widely used regulatory tool to facilitate practice evolution [1,2]. Regulatory (legal) permission to provide new services (such as vaccinations, physical assessment, medication review, independent prescribing for minor ailments, etc.) has often been seen as the most important step and requirement to unleash pharmacists’ potential to provide patient care [3,4]. While it is clear that a permissive regulatory framework and legal guidance is essential for practice change, it is equally clear that simply changing regulations alone will not necessarily incentivize, motivate, or accelerate pharmacists’ adoption of new practice roles [3,5,6,7].

Recent research we completed in Ontario, Canada focused on pharmacists’ perspectives on what supports are required to actually change their practice [8]. This research was undertaken in response to concerns expressed by the regulator, employers, and leaders in professional associations that the pace of practice change and the uptake of new scopes of practice and direct patient care roles by pharmacists was frustratingly slow [3]. A common concern centered on the notion that pharmacists had “asked” for enabling regulation to undertake advanced practice roles, yet once such regulation was promulgated, pharmacists appeared to find other reasons (excuses) for not fully embracing new roles, responsibilities and opportunities to provide patient care [3,4,9]. Through focus group meetings and one-on-one interviews with a total of 46 community pharmacists a model of practice change was developed that identified 9 interdependent supportive factors required (see Table 1). The 9Ps of Practice Change model is premised on the notion that “allowing is not implementing” and that regulatory change does not automatically produce practice change. This model highlights pharmacists’ perspectives on the kinds of educational and practice-related supports needed to provide them with the knowledge, skills, and confidence required to undertake new advanced practice roles aligned with scope of practice expansion.

This previous research [8] focused on pharmacists’ perspectives, but did not necessarily identify specific continuing education (CE) or continuing professional development (CPD)/life-long learning techniques or tactics that could be used or applied. Further research was identified as crucial to translating the 9Ps of Practice Change model into practical educational strategies that would benefit individual pharmacists and enable practice change.

The objective of this research was to identify educational strategies, tactics, and techniques that community pharmacists believe would help support implementation of scope of practice change at the pharmacy level. The 9Ps of Practice Change model was used to guide this research by providing a framework for describing and understanding the psychological and change management dimensions of scope of practice evolution. 

## 2. Materials and Methods 

The objective of this follow-up study focused on pharmacists’ beliefs and perceptions; consequently, a qualitative research methodology was selected, designed to elicit these beliefs and perceptions from participants. Inclusion criteria for this study were modeled on those for the 9Ps of Practice Change study [8] and included the following: (i) licensure as a pharmacist in the province of Ontario for a minimum of 10 years; (ii) active patient-facing practice involving care of and contact with patients for at least 20 h/week in the past 5 years; (iii) self-identification, self-categorization, and self-reporting as a “typical”, “normal” or “average” pharmacist. The focus of this study was on the everyday experience of practice change in community pharmacy; as a result, we specifically excluded individuals who considered themselves to be advanced practitioners, academicians, early adopters of practice change, or practice pioneers.

Participants for this study were recruited from the initial pool of participants involved in the 9Ps of Practice Change study, for convenience and because these individuals had already demonstrated interest in the topic of practice change and willingness to participate in interviews. A total of 46 individuals participated in the original study; invitations were sent by email to all 46 people, and a follow-up reminder was emailed two weeks following the initial invitation to those who did not respond. This invitation outlined the objective of the study, confidentiality provisions, and indicated that participants would be asked to participate in a 30–45 min one-on-one audio taped interview (by phone or in person) exploring their beliefs and perceptions regarding continuous professional development and practice change. Study participation was completely voluntary, and participants were informed that any published data would be anonymized and contain no identifiable data. No remuneration or reimbursement for travel-related expenses was offered for participation in the interview.

The semi-structured interview protocol is provided at Appendix A and was designed to elicit self-reflection and discussion of issues related to professional development. No pilot testing was done with the instrument, though minor amendments were made after the first 3 interviews to enhance efficiency and effectiveness of the protocol. The interviewer maintained extensive field notes to complement audio recordings, and these were incorporated into the data analysis, based on Yin’s case study research design method [10]. After transcription, all data was managed using nVivo v11.1 for Windows, a qualitative data software management program. Random spot checking of transcripts was undertaken (through comparison with original audio recordings) to ensure accuracy. All transcripts were reviewed independently by two coders using inductive methods designed to identify common themes amongst research participants, with the objective of identifying themes where unanimous or near-unanimous agreement occurred amongst all participants. After independent coding, the coders met to discuss and define common themes. Where disagreement in coding occurred, a third coder was available. Content analysis focused on identifying educational strategies, tactics, or techniques to support practice change aligned with the theoretical framework proposed in the 9Ps of Practice Change model. Each “P” of the model served as a node in the initial coding structure to allow for preliminary sorting of data and structuring of the analysis. An iterative coding sequence was used, in which data was transcribed and analyzed as interviews were completed, thereby facilitating use of subsequent interviews for confirmatory purposes. 

This research was approved by the University of Toronto Research Ethics Board. Support for this research was provided through an unrestricted educational grant provided by the Ontario College of Pharmacists, the regulator of pharmacy practice in Ontario, Canada. 

## 3. Results

All 46 participants in the original study were emailed and invited to participate in this follow-up research; of these 20 agreed to participate and 14 declined to participate within the first two weeks. Subsequently, 12 follow-up invitations were emailed to those who had not responded to the initial email; there were no further responses to this follow-up email after two weeks, and recruitment was complete. Those who agreed to participate in the follow-up interview were provided an information sheet regarding study objectives and methods. Follow-up interviews were scheduled; 12/20 agreed to be interviewed by phone, and 8/20 were interviewed in person. All 20 volunteers completed full interviews as scheduled.

Inductive coding and analysis highlighted 7 dominant themes, common across all or most participants, with respect to continuing professional development needs for pharmacists related to scope of practice change:

*(i) A mentoring/coaching style instead of a traditional lecturing/didactic style*

All participants in this study noted that traditional captive continuing education, delivered in a lecture-based or web-based content-intensive format was not appropriate for supporting practice change. While didactic content may be useful for practice change, a didactic teaching approach provided insufficient inspiration or skills development potential to be translated into practice. Instead, a more individualized mentoring-coaching style of interaction was identified by all participants as preferable, as a key learning need for all participants was the need to feel confident and enhance self-efficacy for new roles and responsibilities.

*(ii) In-situ (practice-based) rather than classroom/web-based delivery*

All participants noted that the venue for continuous professional development was also important. While most understood and accepted the practical reality that bulk classroom-based or web-based education was all that was feasible or cost-effective for continuing eduation, all participants stated that ideally, practice-based education should be undertaken within the practice itself (rather than at home or in the classroom/hotel room) as a way to stimulate immediate application. Some participants noted that current pharmacy students benefit greatly from in-situ, experiential education and indicated that this format of delivery would be equally beneficial for experienced practitioners struggling with practice change.

*(iii) A longitudinal, incremental approach to instructional design, rather than a one-off delivery model*

All participants highlighted the limited value of the single topic-driven continuing education model that had dominated professional development for many years. A single 1, 2, or even 3 h lecture or workshop did not support practice change, enhance confidence, or facilitate deep learning. Instead, all participants felt there was value in a staged approach to professional development in which the same topic was addressed over a period of time, to allow opportunities for skill building, reflection, and rehearsal. Many participants noted that practice change is challenging and does not necessarily “take” after the first attempt: an educational model that recognizes the value of longitudinal exposure, while logistically difficult to implement, may be more advantageous for pharmacists’ professional development and learning.

*(iv) “Don’t tell me why I need to change—show me how to change”*

All participants in this study indicated their frustration with the design of most continuing education they attended that overemphasized reasons for practice change rather than actually demonstrating how to change. Most participants indicated they already knew and accepted that practice change in pharmacy was important and necessary; many programs they attended spent much of their time trying to convince attendees of the importance of changing, giving short shrift to the practical issues of implementation of change. Most participants felt that current educational programming operated from the perspective that “we will explain to you why you need to change… but you should be able to figure out for yourself how to change”, and this was not only incorrect, it sometimes came across as dismissive.

*(v) Soft-skills development is under emphasized in most CPD/CE*

All participants noted the abundance of professional development opportunities associated with therapeutic topic areas, such as diabetes management, or asthma control. While valuable, such content did not facilitate practice change because crucial issues related to soft-skills development were rarely discussed and often never the focus of the educational program. Issues related to conflict management, motivational interviewing, effective educational methods, negotiation, and interprofessional collaboration were all identified as crucial to the success of practice change, yet topics such as these were rarely the focus of professional development activity. Therapeutic content, while important, is arguably easier to “look up” on one’s own and facilitated learning of therapeutic content may not be as essential as facilitated learning of such soft skills.

*(vi) Practice*

Of all of the 9Ps of Practice Change, the importance of Practice to support scope of practice evolution was identified by all participants in this study as being not only crucial but almost entirely absent from traditional continuing education programming. Rehearsal and practice of new skills—for example medication reviews—in a safe place with opportunities for coaching and supportive feedback would make a significant difference in uptake of new responsibilities; all participants in this study highlighted their fear of making mistakes, the absence of any systems to provide feedback on performance, and the impact of professional isolation on inhibiting the uptake of new roles and responsibilities. Many participants noted the value of standardized patients in the education of current pharmacy students, and believed that standardized patient-based continuing education would be equally beneficial for pharmacists struggling with practice change.

*(vii) 360-degree feedback*

All participants in this study noted the central importance of supportive, constructive feedback in skills development and in enhancing their confidence to deliver new scope of practice services to patients. All participants also noted that currently, if they ever received feedback on their practice, it rarely came from anyone other than a manager or boss, and was frequently in the form of an annual performance review, or a simple off-handed, unstructured comment. All participants felt it would be valuable to have more structured, holistic feedback provided from a variety of sources, similar to the 360-degree feedback received by many physicians through their professional associations and regulatory bodies. 360-degree feedback involves collection and collation of data from multiple sources (patients, colleagues, other health care professionals, peers, subordinates, etc.) and provides a multidimensional perspective on performance and impact that can be very useful for professional development. The absence of 360-degree feedback systems in pharmacy—and their growing prominence in other fields—suggested opportunities to enhance the quality of continuing professional development programming to support practice change.

Other themes were also identified in this research, but are not presented here as they did not reach the threshold of unanimous or near-unanimous agreement by all participants involved.

## 4. Discussion

The 9Ps of Practice Change model provides a useful theoretical framework for understanding the psychological dimensions of change management unique to pharmacists, but it does not address practical issues related to design and delivery of continuous professional development to support an evolving scope of practice. This research attempted to extend the original research question to incorporate new insights into professional development, in an attempt to provide educational programmers with specific ideas for enhancing the quality and impact of continuing education for practitioners. This approach to understanding pharmacists’ learning needs is unique; we were not able to locate any other similar studies for comparison.

All twenty participants in this research agreed upon the 7 key techniques/tactics highlighted above, each of which introduces new opportunities and challenges for those involved in design and delivery of continuing professional development. Participants in this study were seasoned, experienced practitioners who themselves had personally had to deal with the realities of scope of practice change; their insights into what would help them—and what was less helpful—provide educators and regulators with important information to consider in developing the supports necessary to enable practice change. Traditional continuing education programming has utilized didactic methods (e.g., lectures) focused on one-off therapeutic topics that do not necessarily emphasize development of soft skills. 

These findings provide educational programmers and developers with important insights from pharmacists themselves around the design of continuing professional development activities that may be most supportive of practice change. Of course, these participants were less concerned with or unaware of pragmatic issues (costs, logistics, etc.) associated with delivery of education to practitioners, so some of these desired elements may simply not be feasible in many contexts. Nonetheless, it is sobering and important to note that if practice change is the objective, the pharmacy community will likely need to find better ways than currently exist in providing practitioners with the knowledge, skills, and confidence necessary to embrace new scopes of practice to their fullest.

This study did not aim to identify limitations of current CPD practices and CE delivery models; several participants noted that “traditional” delivery methods (e.g., conference attendance, home-study, web-based modules, pharmaceutical company-sponsored educational events etc.) were still helpful and valuable to their professional development, but all noted that these were likely insufficient if the objective was accelerated practice change. 

Practice change is clearly complex [11] and the lesson of the 9Ps of Practice Change—that “allowing is not implementing”—is important for regulators and educators. Regulatory evolution may eventually produce widespread adoption of new advanced practice roles, but this will take time and change in practice will likely be sporadic, asymmetrical, and unpredictable [8]. Where educational tactics such as those identified by participants in this study can be deployed, it may serve to accelerate the pace of practice change, and ensure it happens in a more uniform manner across the entire profession, rather than in isolated pockets as appears to be the case at the present time. 

It may not be realistic to expect that all—or perhaps any—of these ideas or suggestions from pharmacists could be immediately implemented into CPD/CE activities; importantly, most of these suggestions require a high degree of individual attention to pharmacists, customization of programming, and as a result would likely be cost-prohibitive in many contexts. The challenge going forward will be to identify which of these tactics—or what sub-elements of each of these tactics could perhaps be introduced into widely available programming and to measure what impacts and outcomes occur when they are deployed. Rethinking the profession’s current approaches to CPD and CE, in light of the unique demands associated with scope of practice change, will be required if pharmacy hopes to ensure widespread, rapid diffusion of scope of practice evolution throughout the profession.

## 5. Limitations

This exploratory qualitative study utilized content analysis methods to identify common themes and perspectives of community pharmacists. As the participants were all from one geographical location (Ontario, Canada) generalizability to other jurisdictions should not be assumed. Further, these volunteers had all participated in previous research exploring similar issues, and as a result their views may not be representative of their community. For a qualitative study of this sort, there were a reasonably large number of participants 20 and a threshold of unanimity or near-unanimity suggests a high degree of indicativeness of findings. Still, caution must be exercised in applying findings from this study to other contexts where the experience of practice change may be different.

## 6. Conclusions

For a generation, the profession of pharmacy has advocated for greater responsibilities and independent roles in patient care, commensurate with the education and expertise of pharmacists. Increasingly regulators have agreed and have utilized scope of practice change as an important tool for enabling pharmacists to contribute more fully to patient care, through activities such as vaccinations, medication reviews, and minor ailments programs. Unfortunately, despite having achieved such regulatory milestones, the pace of practice change in community pharmacy appears uneven and slow. This research has identified possible opportunities for continuing professional development to better support practice change through use of different educational tactics and techniques, focused more directly on the site of practice, and pharmacists’ unique and individual learning needs. Identifying methods for incorporating these tactics, or elements of these tactics, into continuing education for pharmacists may help support and accelerate practice change. 

## Figures and Tables

**Table 1:** The 9 pre-requisites (9Ps) of Practice Change in Pharmacy.

Theme	Description
***Permission***	Regulatory context and medical-legal guidance around new scope of practice activities emphasizing legal responsibilities and accountabilities
***Process Pointers***	Explicit procedural guidance for how to implement “expanded scope” into a series of day to day activities or steps in a process. Detailed instructions for how to do specific new activities
***Practice/Rehearsal***	Opportunity to try new psychomotor skills (e.g., vaccination, physical assessment) in a low-stakes, low-risk environment not affecting real patients, and in an environment where they are not being constantly “watched” or scrutinized by others
***Positive Reinforcement***	Encouragement and support to continue to build confidence and skills, rather than constant “second guessing” of personal competence and readiness for expanded scope
***Personalized Attention***	One-size-fits-all educational strategies do not work; individual learning needs vary and a coaching/mentoring style (rather than a teaching/didactic style) is preferred
***Peer Referencing***	Professional isolation reduces confidence and reinforces poor practice habits; opportunities to observe, learn from, and share with peers (not experts of “superstars”) builds confidence and helps identify implementation strategies that work in the real world
***Physician Acceptance***	Concern over interprofessional relationships and “turf battles” can diminish confidence in new skill areas; support from physicians for new activities (especially those that used to be performed by physicians) is essential to motivating pharmacists
***Patients’ Expectations***	As patients become progressively more aware of pharmacists’ expanding scope, they place positive pressure on pharmacists to “step up” and in turn their gratitude and appreciation for the pharmacist’s help builds confidence and enhances willingness to expand practice
***Professional Identity***	Historical role of pharmacist as someone who “follows orders” or only dispenses is at odds with scope of practice expansion; an evolving professional self-identity as an independent clinician, responsible decision-maker, and interprofessional collaborator is necessary

## Appendix A

**Table A1:** Semi-Structured Interview Protocol Excerpts and Sample Data Coding.

Interview Question	Sample Transcript Excerpt	Coding
What kinds of learning do you do to keep up to date in practice?	“The usual—I go to conferences, attend [drug company dinners] that sort of stuff. Honestly, I don’t know how useful it is though—I mean after an hour in a hotel ballroom, just listening to a lecture, what are you really supposed to take away from that?” (Female 47 yo)	**Traditional Lecture unhelpful** **Large group didactic setting unhelpful**
What do you think would help you with implementing practice change?	“Stop just telling us why we need to change—honestly sometimes I feel like some of these speakers, some of the leaders actually, think we all must be idiots or something. Of course we know why pharmacy should needs to change. Actually help us by showing us what we’re supposed to do in order to change.” (Male 52 yo)	**Don’t tell me why to change, show me how to do it**
What specific areas do you feel you would benefit most from learning more about, in order to change your practice?	“It’s not just me, I’m sure, but I think all of us, all pharmacists need more training in what they call the soft skills. You know, how to manage conflict, how to talk to patients and doctors—especially talking to doctors. Not to feel nervous or overwhelmed but I don’t know more confident?” (Male 51 yo)	**Soft skills training needed**
Have you heard of, or experienced, any kind of continuing education or continuing professional development you think would be particularly helpful for you or other pharmacists to support practice change?	“These students have it good, you know. I really like the emphasis now on [in-practice] experiential education. Learning in a pharmacy is honestly the best way to do it. I really wish this was something available to pharmacists, older pharmacists, like me, instead of just the old lectures, or webinars, which I don’t think really work as well.” (Female 39 yo) “I know in school, young pharmacists now spend a lot of time with those standardized patient actors. I think that’s absolutely excellent—what a great chance to you know practice, try out different communication or counselling, get feedback and it’s not for real, it doesn’t involve a real patient if you make a mistake. This is something pharmacists could really really use, the chance to practice with actors and learn that way.” (Female 44 yo)	**In-practice/experiential learning valuable** **Traditional Lecture unhelpful** **Practice using standardized patient eduators**
Can you describe how feedback or assessment helps you to improve and influences practice change for you?	“Doing those ridiculous 20-question quizzes after you do an on-line course, to prove you learned something—that’s not at all helpful, just a hoop to jump through honestly. I know the [physicians] I work with, they have this 360 degree feedback system, where someone gathers feedback from their patients, their nurses, their staff—I’ve actually been contacted a few times to give feedback about physicians too. Most pharmacists do this these days with their docs. I think that’s awesome. It’s all anonymous and they really get to discover what other people think of them, their practice as a physician. We desperately need this kind of system in pharmacy too, so we know what others are thinking of the job we are doing.” (Male 50 yo)	**360-degree feedback system to facilitate practice change**
For you personally, what would be most helpful in helping you to implement new scope of practice roles?	“For me—I don’t think I’m probably the only one who says this—it’s really hard to learn about this stuff by, I don’t know, reading a journal article, or going to a conference. It would be great if there was someone there, in my store, right beside me to guide me, give me pointers, correct me early on if I’m doing something wrong. I don’t think it would take a long time, but you know, a regular lecture it’s not the same as having someone right there beside you.” (Female 41 yo)	**Traditional lecture unhelpful** **Coaching/Mentoring support model** **In-practice/experiential learning valuable**
What are some of the difficulties you’ve experienced in implementing expanded scope of practice?	“I try to keep up with my CE, you know, but I don’t think it works. Too much information coming at you too fast so you don’t retain anything, and honestly it’s forgotten by the next day. It would be better to have some kind of reinforcement—the same topic on several different days or occasions, with less intense content all at once. You know, so you can absorb something, try it, get confident, then learn something new to add on to it after that.” (Female 51 yo) “It sounds strange, but the pharmacy part—the drugs I mean—that’s the easiest for me, for most people. You can look it up, right? What’s hard are all the people interactions now—patients, supervising technicians, talking with doctors. There’s a lot more demands in that area and we weren’t trained for that. That’s what people like me need to help us with expanded scope, not more lectures on diabetes or asthma.” (Male 46 yo)	**Longitudinal learning model rather than intensive one-off lecture** **Soft skills training needed**
